# Poster Session I - A165 5-FU RESISTANT COLON CANCER ORGANOIDS, A GREAT TOOL TO DEMYSTIFY THE INTERACTION BETWEEN CANCER STEM CELLS AND CANCER-ASSOCIATED FIBROBLASTS

**DOI:** 10.1093/jcag/gwaf042.165

**Published:** 2026-02-13

**Authors:** M Sedeuil, E Grenier, V Giroux

**Affiliations:** Cell biology, Universite de Sherbrooke Faculte de Medecine et des Sciences de la Sante, Sherbrooke, QC, Canada; Cell biology, Universite de Sherbrooke Faculte de Medecine et des Sciences de la Sante, Sherbrooke, QC, Canada; Cell biology, Universite de Sherbrooke Faculte de Medecine et des Sciences de la Sante, Sherbrooke, QC, Canada

## Abstract

**Background:**

Colon cancer is the 2^nd^ deadliest cancer worldwide. The main treatment combines surgery with 5-fluorouracil (5-FU) chemotherapy. However, approximately 70% of patients will relapse due to a loss of sensitivity to treatment, known as chemoresistance. Over the past decade, studies have revealed the importance of two tumor components: cancer stem cells (CSC) and cancer-associated fibroblasts (CAF). While their individual contribution to resistance has been well studied, a deeper understanding of their respective modulation and interactions during resistance remains essential.

**Aims:**

Identify the specific modulation occurring in CSC and the impact of the crosstalk between CSC and CAF during the development of 5-FU resistance in colon cancer.

**Methods:**

To achieve this goal, we established two models of colon cancer organoids resistant to 5-FU. To do this, we exposed our parental organoids with cyclic 5-FU treatment, mimicking clinical practice.

**Results:**

We validated the establishment of resistance by showing an important increase in IC50 for both resistant lines, using the WST1 test. We also demonstrated an increase in CSC (CD133^+^) and clonogenic capacity in resistant organoids. Preliminary proteomic profiling of the sorted CSC in parental organoid lines, treated with acute 5-FU treatment, suggest the importance of GTPase signaling in the response to 5-FU. Similar experiments are being conducted on CSCs from resistant organoids to identify key pathways involved in 5-FU resistance. On the other hand, our findings indicate that the conditioned media of CAF cultures increases the growth capacity of organoids and CSC proportion, highlighting the necessity for further exploring the interaction between these two tumor components. To this purpose, ALI coculture have been successfully established from colon cancer organoids and CAF which will allow us to functionally address the impact of CAF on CSC. To do so, secretome and surfaceome analysis will be performed.

**Conclusions:**

In summary, our results show that cyclic 5-FU treatment was the appropriate method to induce 5-FU resistant in our organoids. Our study on CAF conditioned media demonstrates that the interaction between tumor cells and CAF influence CSC capacities. The ALI culture system supports the growth of both CAFs and colon cancer organoid cells. The goal of this project is to improve our understanding of the interactions between CAFs and CSCs in the context of resistance, paving the way for new targeted therapies.

**Funding Agencies:**

CIHRCancer research society

